# Mesoporous Silica and Titania-Based Materials for Stability Enhancement of Polyphenols

**DOI:** 10.3390/ma14216457

**Published:** 2021-10-28

**Authors:** Mioara Prundeanu, Ana-Maria Brezoiu, Mihaela Deaconu, Gratiela Gradisteanu Pircalabioru, Daniel Lincu, Cristian Matei, Daniela Berger

**Affiliations:** 1 Department of Inorganic Chemistry, Physical-Chemistry and Electrochemistry, University “Politehnica” of Bucharest, 1–7 Gheorghe Polizu Street, 011061 Bucharest, Romania; mioara_prundeanu@yahoo.com (M.P.); anamaria.brezoiu@gmail.com (A.-M.B.); mihaela_deaconu@yahoo.com (M.D.); daniel.lincu1113a@gmail.com (D.L.); cristian.matei@upb.ro (C.M.); 2 Research Institute of the University of Bucharest (ICUB), Division of Earth, Environmental and Life Sciences, 91–95 Splaiul Independenței, 050095 Bucharest, Romania; gratiela.gradisteanu@icub.unibuc.ro; 3 “Ilie Murgulescu” Institute of Physical Chemistry, Romanian Academy, 202 Splaiul Indepedentei, 060021 Bucharest, Romania

**Keywords:** common sage, polyphenolic extract, ultrasound-assisted extraction, encapsulation, mesoporous titania nanoparticles, titania-ceria composite

## Abstract

To improve phytochemical stability, polyphenolic extracts prepared from *Salvia officinalis* L., which is a valuable source of phytocompounds with health benefits, were embedded into mesopores of silica, titania, or titania-ceria materials. Ethanolic and hydroalcoholic extracts were prepared by conventional, microwave- or ultrasound-assisted extraction. The influence of the extraction conditions on chemical profile, radical scavenger activity (RSA), and antimicrobial potential of the extracts was assessed. The extracts were characterized by spectrophotometric determination of total polyphenols, flavonoids, chlorophyll pigment contents, as well as RSA. A reverse phase HPLC- PDA analysis was performed for the identification and quantification of extract polyphenols. The extract-loaded materials exhibited an enhanced RSA compared to the free extract after several months of storage, resulting in better polyphenol stability over time following embedding into a mesoporous matrix. Selected extracts free and embedded into mesoporous support were tested against *Pseudomonas aeruginosa* ATCC 27853, *Escherichia coli* ATCC 25922, and *Staphylococcus aureus* ATCC 25923; the best antimicrobial activity was obtained for *S. aureus*. A slight improvement in antimicrobial activity was observed for the ethanolic extract prepared by ultrasound-assisted extraction following embedding into the TiO_2_ matrix compared to MCM-41 silica due to the support contribution.

## 1. Introduction

Polyphenols are an important class of micronutrients found in plants, like vegetables, herbs, fruits, and cereals; they are not synthesized in animal organisms [1,2]. They have various chemical structures and, depending on the number of aromatic rings in their chemical formulas, are categorized as flavonoids, phenolic acids, stilbenes, or lignans [3]. Medicinal plants are a good source of the polyphenolic compounds [4,5], and as such, are widely used in traditional medicine. Their extracts have great potential as antioxidants, anti-inflammatory and antimicrobials for food, cosmetics, or nutraceuticals [6].

The recovery of natural compounds from medicinal plants can be optimized by careful consideration of the extraction parameters, such as solvent/plant ratio, temperature, contact time, type of solvent, microwaves (MW) [7] or ultrasounds irradiation (UAE) [8], or the use of a high voltage electrical discharge [9,10,11,12,13]. For instance, a higher temperature decreases the viscosity and surface tension of the extraction medium, helping the solvent to penetrate the plant matrix and improving the wetting of vegetal material, respectively, leading to higher extraction yield [14]. For the extraction of polyphenols from plants, various solvents, like methanol, ethanol, acetone, water [15], hexane, ethyl-acetate [16] etc. have been used. The selection of a solvent for the extraction of phenolic compounds is based on the solute polarity, i.e., a solvent of similar polarity to the solute dissolves it effectively [17,18]. For instance, to obtain polyphenolic extracts from dried common sage leaves, Dent et al. used 30%, 50% and 70% aqueous solution of ethanol or acetone and temperatures of either 60 °C or 90 °C. They reported the best efficiency for 30% aqueous solution of ethanol at 60 °C [19]. Extraction assisted by high voltage electrical discharge causes the electroporation of the plant membrane, which increases the release of polyphenolic compounds; hence, a 2.1 times higher total polyphenols content in sage extract was obtained compared to conventional extraction [9]. Microwaves-assisted extraction has led to a more efficient recovery of phenolic compounds from common sage than conventional methods due to the higher radical scavenger activity (RSA) of the MW extract compared to the conventional one obtained in the same conditions [7]. Ramic et al. reported that the extraction of polyphenols from Aronia melanocarpa by-product from filter-tea was accelerated by sonication, with ultrasonic power being varied from 72 to 216 W and extraction time and temperature in the range of 30–90 min. and 30–70 °C, respectively. Based on a statistical analysis of data, they showed that temperature was the most influential factor in the extraction. High temperature and time enhanced phenolic, flavonoid and proanthocyanidin content and reduced anthocyanin amount in the extracts; this was attributed to the lower resistance to thermal degradation of the latter compared to other phytocompounds [20].

*Salvia officinalis* (common sage) from the *Lamiaceae* family, native to the Mediterranean region, is a valuable source of phytochemicals with antioxidant, anti-inflammatory, and antimicrobial properties [21,22,23]. The health benefits of common sage extracts depend on their chemical composition. For example, Dal Prá et al. [24] showed that the anti-inflammatory effect of sage extracts is associated with a large amount of ursolic, rosmarinic, caffeic, and oleanolic acids, while Veira et al. [22] reported even higher anti-inflammatory activity of *Salvia officinalis* extracts than diclofenac or salicylic acid. Studies have supported the evidence that caffeic acid and its derivatives, i.e., rosmarinic, salvianolic, sagerinic acids, which are present in many types of *Salvia* extracts, are effective in hepatoprotection and protective against heart ischemia-reperfusion [25]. Also, in vitro animal and preliminary human studies have shown that polyphenols from *Salvia* plants have promising effects in terms of the enhancement of cognitive activity and prevention of neurodegenerative diseases [26].

Despite their health benefits, the instability of polyphenolic compounds limits their use. To prevent the degradation of phytocompounds during industrial processing, which depend on pH, temperature, light exposure etc., a solution can be their encapsulation in different matrices such as chitosan [27], alginate [28], liposomes [29], pristine and functionalized mesoporous silica [30,31] etc.

The aim of this research was to improve the stability of selected polyphenolic extracts through encapsulation into mesopores of titania, titania-ceria composite or silica materials. Also, the influence of the extraction conditions on the chemical profile, radical scavenger activity and antimicrobial potential of common sage polyphenolic extracts was assessed. The radical scavenger activity and antimicrobial potential of the materials containing embedded extract, as well as the recovery of polyphenols from mesoporous matrices in phosphate buffer solution, were evaluated. Both mesoporous silica and titania-based materials proved to be biocompatible, showing no significant toxicity on different cell lines if the dose was limited [32,33,34]. These compounds are already used as additives in the pharmaceutical and cosmetics industry, being considered safe by the US Food and Drug Administration [35,36]. Due to their large pore volume, mesoporous silica and titania are good hosts for phytocompounds. Moreover, titania nanoparticles (TiO_2_ NPs) exhibit bactericidal activity under UV irradiation [37]. They are negatively charged in neutral and basic solutions, showing low bactericidal activity, but in acidic media, TiO_2_ NPs become positively charged, allowing them to penetrate the bacterial membrane inducing their damage [38]. In the case of rare earth doped titania, the dopant ions are effective in suppressing the recombination of electrons and holes in TiO_2_ NPs. Kasinathan et al. reported that cerium doped titania exhibited bactericidal activity, especially against Gram-positive bacteria, which may be attributed to its strong oxidation activity and superhydrophilicity [39].

## 2. Results and Discussion

### 2.1. Characterization of Common Sage Polyphenolic Extracts by Spectrometric Measurements

Ethanolic and hydroalcoholic (ethanol-water 1/1 *v*/*v*) polyphenolic extracts from *Salvia officinalis* L. dried leaves were prepared using different plant/solvent weight ratios, at 80° or 50 °C by conventional, microwave (So(MW)-2) or ultrasound-assisted (So(US)-1) extraction in three extraction stages after a maceration step lasting 20 h. The extract yield, total polyphenol, flavonoid and chlorophyll pigment content, as well as radical scavenger activity (RSA), determined by spectrochemical methods, of the polyphenolic extracts prepared in different conditions are presented in Table 1. The use of an ethanol-water mixture (1:1 *v*/*v*) as a solvent yielded superior recovery of phytocompounds from common sage compared to absolute ethanol. Thus, the highest yield (31.7%) was obtained for the hydroalcoholic So(Conv)-3 extract prepared at 50 °C, followed by the other hydroalcoholic extracts, So(Conv)-5 (24.5%) and So(MW)-2 (19.9%) obtained at reflux, while the lowest efficiency was observed for the ethanolic So(Conv)-6 extract (8.2%) prepared at 50 °C. It was observed that the application of low intensity ultrasound (maximum input power of 320 W) during the extraction process, that usually do not alter the state of the vegetal material but decrease the time of the extraction, correlated with higher yield (13.0% for So(US)-1) in natural compounds compared to the conventional method (8.2% for So(Conv)-6). In agreement with literature data, ultrasound-assisted extraction (UAE) led to enhanced recovery of natural compounds as a result of the breaking down of plant tissues into the solvent containing the vegetal material [40].

The total polyphenol content (TPC) of the prepared common sage extracts determined through the Folin Ciocalteu method as gallic acid equivalents was in the range of 129.20–192.81 mg GAE/g extract (11.30–46.11 mg GAE/g plant) (Table 1). The richest extract in terms of polyphenolic substance content was that obtained by UAE, So(US)-1, which also had a higher amount of polyphenolic compounds than previously reported for common sage extracts obtained at reflux in absolute ethanol or 4/1 (*v*/*v*) ethanol/water mixture by either conventional or MW-assisted extraction [7]. The hydroalcoholic extracts had a higher content of polyphenols than ethanolic extracts, which is in agreement with literature data [22]. For instance, the hydroalcoholic So(Conv)-3 extract prepared at 50 °C had the highest total polyphenol content (46.11 mg GAE/g plant), while both hydroalcoholic So(MW)-2 and So(Conv)-5 obtained at reflux exhibited lower TPC values, i.e., 33.66 mg GAE/g plant and 40.49 mg GAE/g plant, respectively. The So(Conv)-3 extract had an even higher content than that reported by Nutrizio et al. (42.13 ± 1.24 mg GAE/g plant) for the sage hydroalcoholic extract obtained by high voltage electrical discharge-assisted extraction [9]. Unlike the hydroalcoholic extracts, in the case of the ethanolic extracts, an increase of the temperature in the extraction process favored the recovery of polyphenols from *Salvia officinalis* leaves (18.02 mg GAE/g plant for So(Conv)-4 against 11.30 mg GAE/g plant for So(Conv)-6), though the So(Conv)-6 extract prepared at 50 °C was richer in phenolic compounds (138.11 ± 2.45 mg GAE/g extract) than So(Conv)-4 sample (129.20 ± 5.59 mg GAE/g extract). The TPC values of these extracts when expressed per 1 g of vegetal material were lower than those previously reported by us for the *Salvia officinalis* extract prepared by conventional extraction at reflux in 4/1 (*v*/*v*) ethanol/water mixture (61.98 mg GAE/g plant) [7]. The TPC values of our *Salvia officinalis* extracts were higher than those reported for 70/30 (*v*/*v*) ethanol-water extracts prepared either by conventional or ultrasound-assisted extraction (61.3–79.6 mg GAE/g extract) [41], 80% aqueous methanolic extracts obtained by ultrasounds-assisted extraction (2.80 mg/g plant) [42], and methanolic extracts (63.9–93.8 mg/g extract) [43], as well as aqueous *Salvia officinalis* extract (158.9 ± 38.0 mg GAE/g extract), but only those prepared by nonconventional methods and the hydroalcoholic So(Conv)-5 extract [5], which presented TPC values lower than those reported by Veira et al. for hydroalcoholic and ethanolic extracts prepared through Soxhlet extraction using a 1/10 g plant/mL solvent ratio (685.2 ± 6.6 mg/g extract) [22].

The total flavonoid content (TFC) as quercetin equivalents (QE) determined using the aluminum chloride colorimetric method was in the range of 15.42–36.98 mg QE/g extract (1.26–7.96 mg QE/g plant) (Table 2). The ethanolic So(Conv)-4 extract was the richest in flavonoids when the TFC value was expressed per gram of extract (36.98 ± 1.22 mg QE/g extract), while the hydroalcoholic extract So(Conv)-3 prepared at 50 °C had the highest TFC value per gram of plant (7.96 ± 0.16 mg QE/g pant). These TFC values were slightly higher than those reported by us for the ethanolic and hydroalcoholic (4/1 (*v*/*v*) ethanol/water) *Salvia officinalis* extracts (13.56–25.03 mg QE/g extract) [7], but close to those obtained for ethanolic extracts by Duletic-Lausevic et al. (27.30 ± 8.48 mg/g extract) [44]. Interestingly, the extracts prepared at 80 °C, either in absolute ethanol or in water-ethanol mixture, had very similar TFC values (5.16–5.78 mg QE/g plant), while in the case of the extracts obtained at 50 °C, the ethanol-water mixture was a better solvent for flavonoid release from vegetal material (7.96 mg QE/g plant for So(Conv)-3) in comparison with ethanol (3.17 ± 0.03 mg QE/g plant and 1.26 ± 0.01 mg QE/g plant for So(US)-1 and So(Conv)-6, respectively).

The total chlorophyll pigment content (TChC) of the common sage extracts was in the range of 0.53–12.86 mg Ch/g, with ethanolic extracts having a higher amount than the hydroalcoholic ones (Table 1), in agreement with previous results [7,45]. The UAE process favored the recovery of chlorophyll pigments, with the So(US)-1 extract having a much higher TChC value than even the other ethanolic extracts discussed here, i.e., So(Conv)-4 and So(Conv)-5, or the common sage ethanolic extract obtained through MW-assisted extraction [7], probably because ultrasound can destroy the cell wall of plants. Therefore, it was observed that the use of a less polar solvent, i.e., ethanol, as well as ultrasound-supplementary energy, led to a better recovery of the chlorophyll pigments.

In conclusion, the use of the water-ethanol mixture as solvent favored both polyphenol and flavonoid extraction. A temperature of 80 °C for the extraction process improved the recovery of phenolic substances from common sage leaves when ethanol was used as the solvent; this was not the case with the water-alcohol mixture.

### 2.2. Radical Scavenger Activity of Polyphenolic Extracts

The radical scavenger activity (RSA) against 2,2-diphenyl-1-picrylhydrazyl (DDPH^•^) and 2,2′-azino-bis(3-ethylbenzothiazoline-6-sulfonic acid)diammonium salt (ABTS^•+^) of polyphenolic compounds derived from dried *Salvia officinalis* L. leaves in different conditions was assessed and expressed as Trolox equivalents (TE) per gram of extract; see Table 1. The RSA values for the polyphenolic extracts were in the range of 113.36–249.07 mg TE/g and 98.22–298.34 mg TE/g extract in the case of the ABTS assay and DPPH method, respectively. Mostly higher RSA values were observed when the ABTS method was applied, especially for the hydroalcoholic extracts, a phenomenon which may be attributed to their higher phenolic substances content, in comparison with the ethanolic extracts. Previously, we showed that the ethanolic extract obtained by MW-assisted extraction exhibited better antioxidant capacity than the conventional extract prepared under the same conditions (232.71 ± 8.23 mg TE/g extract against 96.81 ± 3.63 mg TE/g extract) [7]. In this study, the hydroalcoholic So(MW)-2 presented a slightly lower RSA (232.32 ± 0.73 mg TE/g extract against ABTS^•+^ radicals and 211.86 ± 4.45 mg TE/g extract against DPPH^•^) than the So(Conv)-5 extract prepared under the same conditions (249.07 ± 6.93 mg TE/g extract-ABTS and 268.11 ± 11.22 mg TE/g extract-DPPH) in the case of both assays, but a higher RSA than the hydroalcoholic So(Conv)-3 extract obtained at 50 °C (215.20 ± 4.22 mg TE/g extract) when ABTS assay was used. Regarding the ethanolic So(US)-1 extract, a better ability to neutralize ABTS^•+^ radicals (245.68 ± 6.28 mg TE/g extract) than DPPH^•^ radicals (201.29 ± 16.36 mg TE/g extract) was observed. A temperature of 80 °C applied during the conventional extraction process led to a higher ability to neutralize ABTS^•+^ radicals of both hydroalcoholic and ethanolic extracts compared to 50 °C, in accordance with the data reported by Sotiropoulou et al. [46]. The IC50 values (the concentration which determines the inhibition of 50% of DPPH^•^ radicals from solution) of the polyphenolic extracts determined by DPPH assay using three concentrations from the linearity domain are listed in Table 1; the lowest IC50 value was observed for the So(Conv)-3 extract.

### 2.3. Chemical Profile of Common Sage Extracts

**Figure 1:**
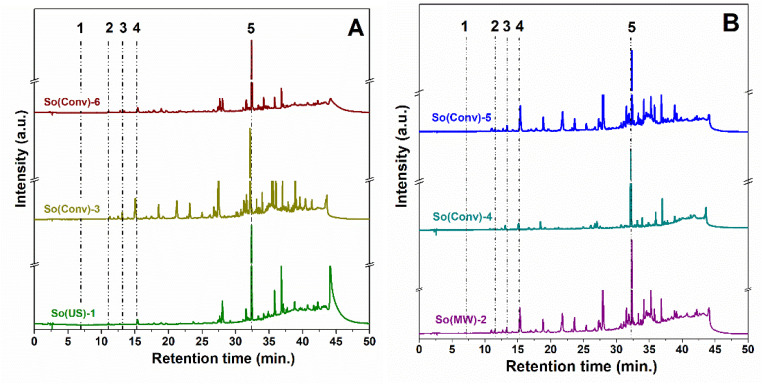
HPLC-PDA chromatograms for ethanolic (**A**) and hydroalcoholic extracts (**B**) The identified compounds are: 1-protocatechuic acid, 2-caftaric acid, 3-chlorogenic acid, 4-caffeic acid, 5-rosmarinic acid.

A reverse phase HPLC–PDA analysis led to the identification and quantification of up to five phenolic compounds from 23 available standards based on their retention time and the similarity of UV spectra with those of standard substances. Chromatograms of common sage polyphenolic extracts are presented in Figure 1. All extracts contained rosmarinic acid (5.673–35.335 mg/g extract) as the most abundant compound, as well as caffeic acid (0.174–2.494 mg/g extract) and chlorogenic acid (0.094–1.028 mg/g extract), while protocatechuic acid (0.235–0.571 mg/g extract) was present only in hydroalcoholic extracts (Figure 1B), and caftaric acid (0.587–0.760 mg/g extract) in the extracts prepared through nonconventional extraction and So(Conv)-5 (Table 2).

Rosmarinic acid was more efficiently recovered in absolute ethanol than in 50% aqueous ethanolic solution, especially when UAE was used, with the highest content (35.335 ± 0.000 mg/g extract) being observed for So(US)-1 extract (Table 2). The extraction temperature does not seem to influence the content of the polyphenolic extracts in rosmarinic acid compared to hydroalcoholic So(Conv)-3 (22.877 ± 0.004 mg/g extract) and So(Conv)-5 (20.542 ± 0.009 mg/g extract). However, the common sage extracts reported herein had a lower rosmarinic acid content than our previously reported extracts obtained by conventional extraction at reflux, in ethanol or 4/1 (*v*/*v*) ethanol/water (41.600–49.975 mg/g extract) [7].

The extraction of caffeic acid from *Salvia officinalis* dried leaves favors the use of a water-ethanol mixture as an extraction solvent, with all hydroalcoholic extracts having similar contents, i.e., ranging from 2.175 mg/g extract to 2.632 mg/g extract with a slightly higher yield for the extracts obtained at reflux, So(MW)-2 and So(Conv)-5, compared to the So(Conv)-3 extract (Table 3).

Regarding the chlorogenic acid present in all extracts, the highest amount was identified in the So(Conv)-4 extract (1.028 ± 0.001 mg/g extract), while the other ethanolic extracts prepared at 50 °C had lower contents (0.094–0.330 mg/g extract) (Table 3). All hydroalcoholic extracts had a similar quantity of chlorogenic acid (0.0675–0.828 mg/g extract), with a slightly higher amount being observed in the So(Conv)-3 extract, prepared at 50 °C.

Caftaric acid was detected in the extracts prepared by UAE, ethanolic So(US)-1 (0.760 ± 0.000 mg/g extract), and hydroalcoholic extracts So(MW)-2 (0.587 ± 0.001 mg/g extract) and So(Conv)-5 (0.753 ± 0.005 mg/g extract) obtained at reflux. Previously, we observed that the use of an ethanol-water mixture as the extraction solvent favored the extraction of caftaric acid in comparison with absolute ethanol [7]. Based on these and previously reported data, the application of UAE or an increase in temperature led to the enhancement of the caftaric acid recovery.

### 2.4. Antibacterial Activity Assessment of Polyphenolic Extracts

The ethanolic So(US)-1 and So(Conv)-6 extracts were chosen for testing on three standard bacteria: *Escherichia coli* ATCC 25922, *Pseudomonas aeruginosa* ATCC 27853 and *Staphylococcus aureus* ATCC 25923, because So(US)-1 had the highest polyphenolic compounds content while So(Conv)-6 presented the lowest amount of phytocompounds. The minimum inhibitory concentrations (MICs) of the *Salvia officinalis* L. extracts are presented in Table 3. Slight better antibacterial activity was noticed against *S. aureus* ATCC 25923 compared to *E. coli* ATCC 25922 or *P. aeruginosa* ATCC 27853 strains. *Pseudomonas aeruginosa* and *Escherichia coli* are common pathogens which are responsible for prolonged infections because of their ability to form biofilms. It is notable that the minimum biofilm eradication concentration (MBEC) of the common sage extracts against the two Gram-negative bacteria is lower than the corresponding MIC, i.e., two and four times lower against *P. aeruginosa* and *E. coli,* respectively. Although the polyphenolic compound contents of the tested extracts were different, their MIC and MBEC against tested strains were the same, except in the case of *S. aureus*. The MIC value against *S. aureus* was lower for So(US)-1 extract (437.5 µg/mL) than for So(Conv)-6 (875 µg/mL).

### 2.5. Characterisation of Mesoporous Supports

To encapsulate the chosen polyphenolic extracts, three inorganic materials, i.e., mesoporous titania and titania-ceria nanoparticles obtained by original methods based on the sol-gel technique, as well as commercial MCM-41 silica, were employed. As inorganic matrices, we chose titania nanoparticles, because they provide a versatile platform for various biologically active molecules due to their low toxicity, biocompatibility and chemical stability. Additionally, when doped or associated with ceria nanoparticles (CeO_2_ NPs), enhanced photostability is achieved, making them applicable to radiotherapy image diagnoses and photodynamic therapy [47]. Recently, CeO_2_ NPs were described as nanozymes (nanomaterials with enzyme-like activity) that mimic the behavior of natural antioxidative enzymes [48]. Thus, CeO_2_ NPs have been extensively tested as an antioxidant compound to treat neuronal disorders or autoimmune degenerative diseases. Yu et al. reported a robust superoxide dismutase and catalase mimetic activities for CeO_2_ NPs distributed into a metal organic framework, allowing them to neutralize the damage produced by reactive oxygen species generated by new-born neutrons [49]. Also, hollow CeO_2_ NPs functionalized with chitosan and ZM241385 have been employed as nanocarriers for pilocarpine in an ophthalmic nanoformulation to prevent glaucoma progression [48].

The inorganic matrices used for the embedded common sage extracts were investigated by wide-angle powder X-ray diffraction to determine their structure, while FTIR spectroscopy and thermal analysis were applied to assess the removal of the template agent used in their synthesis. Additionally, N_2_ adsorption-desorption isotherms were used to determine textural features (specific surface area, total pore volume and the average pore diameter), and SEM and TEM were applied to investigate their morphology.

**Figure 2:**
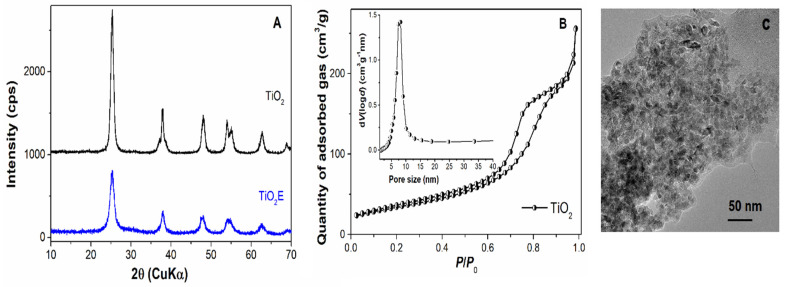
Titania nanoparticle characterization: wide–angle XRD patterns of TiO_2_E and TiO_2_ (**A**); N_2_ adsorption–desorption isotherm recorded at 77 K of TiO_2_ and inset, the corresponding pore size distribution curve (**B**); TEM image of TiO_2_ (**C**).

In the case of the titania material, wide-angle XRD analysis showed the formation of an anatase phase with tetragonal symmetry (ICDD 21-1272) after solvothermal treatment performed at 100 °C for 24 h, TiO_2_E (Figure 2A). Because of the strong interactions between titania nanoparticles and the copolymer, Pluronic P123 (used as a template agent), the washing step followed by Soxhlet extraction in ethanol for 24 h were not enough to completely remove the structure directing agent, as revealed by FTIR spectroscopy (see Supplementary Materials, Figure S1A). Therefore, a calcination step at 400 °C for 3 h was required to obtain pure TiO_2_ NPs with anatase structure (see Figure 1B–curve black). The calcination step led to an increase in the crystallite size, as determined by Rigaku’s PDXL software based on Scherrer’s equation from (1 0 1) Bragg reflection (2*θ* = 25.29 °), from 7 nm for TiO_2_E to 10 nm for TiO_2_ calcined at 400 °C.

The N_2_ adsorption-desorption isotherm of the titania material, specific for mesoporous materials, exhibited hysteresis at *P/P*_0_ > 0.6 (Figure 2B), which corresponded to a narrow, unimodal pore size distribution curve (Figure 2B–inset). The textural parameters, the specific surface area determined by BET method, *S_BET_*, total pore volume measured at *P/P*_0_ = 0.99, *V_p_*, and the average pore diameter calculated with Barrett Joyner Halenda model, *d_BJH_*, for TiO_2_ support are presented in Table 4. The morphology of the TiO_2_ NPs was investigated by TEM. The titania sample, prepared by the sol-gel method assisted by solvothermal treatment, comprised small, uniform sized polyhedral nanoparticles with average dimension of 10 nm (in agreement with the crystallite size computed by Scherrer’s equation from the XRD pattern) and forming intergranular pores between nanoparticles (see Figure 2C). In the FTIR spectrum of TiO_2_ obtained at 400 °C, only the characteristic vibrations of titania, i.e., 670 cm^−1^ (ν_Ti-O_), 478 cm^−1^ (ν_Ti-O-Ti_) and 3000–3600 cm^−1^ (ν_Ti-OH_), can be observed, as well as the band of physically adsorbed water molecules, δ_HOH_, at 1645 cm^−1^ [50] (see Supplementary Materials, Figure S1A). The DTA-TG analysis (see Supplementary Materials, Figure S1B,C) showed that the thermal treatment at 400 °C for 3 h completely removed the copolymer, as no effect was recorded on the DTA curve of the calcined titania sample (see Supplementary Materials, Figure S1B).

**Figure 3:**
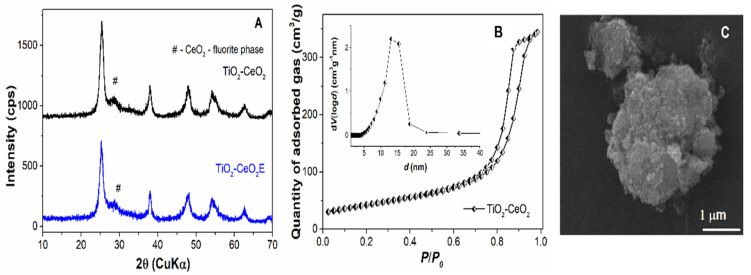
Titania–ceria composite characterization: wide-angle XRD patterns of TiO_2_-CeO_2_E and TiO_2_-CeO_2_ (**A**); N_2_ adsorption–desorption isotherm recorded at 77 K of TiO_2_-CeO_2_ calcined at 450 °C, 5 h and inset showing the corresponding pore size distribution curve (**B**); SEM image of TiO_2_-CeO_2_ composite material (**C**).

In the case of the titania-ceria composite, the XRD results showed the formation of a crystalline material, TiO_2_-CeO_2_ precursor (the material recovered after the ageing treatment of the reaction mixture), with a fluorite phase for ceria and the anatase structure of titania. Thermal treatment of the TiO_2_-CeO_2_ precursor at 450 °C for 5 h preserves the anatase and fluorite phases (Figure 3A). Interestingly, although the calcination temperature in the case of the titania-ceria composite was higher than that of the TiO_2_ sample, it led to a very small increase in the crystallite size determined from the (1 0 1) Bragg reflection of the anatase phase, i.e., from 7 nm to 8 nm, in contrast to the titania sample. The cerium ions suppressed the increase of crystallite size. In the FTIR spectrum, one can observe very intense bands centered at 667 cm^−1^ and 484 cm^−1^, which may be attributed to the metal-oxide bonds (Supplementary Materials, Figure S2A) that had been shifted, in comparison with the Ti-O vibrations observed in the case of TiO_2_ NPs (713 cm^−1^ and 460 cm^−1^) (see Supplementary Materials, Figure S1A). The characteristic vibration band of the physiosorbed water molecules at 1635 cm^−1^ was more intense for the titania-ceria composite than in the titania material, probably because of a higher concentration of oxygen vacancies in the former, favoring the adsorption of water [39].

The N_2_ adsorption-desorption isotherm of the titania-ceria material was shown to be type IV, according to IUPAC classification, with a unimodal pore size distribution curve centered at a higher pore diameter, (13.18 nm) than for TiO_2_ (7.43 nm) (see Figure 3B). Usually, among the textural parameters determined from N_2_ adsorption-desorption isotherms, the most important is the total pore volume, which is directly proportional with the amount of phytocompounds that can be hosted on support mesopores. For the titania-ceria composite material, the specific surface and total pore volume had higher values (150 m^2^/g and 0.54 cm^3^/g, respectively) in comparison with the titania support (124 m^2^/g and 0.26 cm^3^/g, respectively) (see Table 4). The SEM investigation of the titania-ceria composite material revealed the formation of primary nanoparticles which agglomerated in aggregates with different sizes and shapes (Figure 3C). The Ti/Ce molar ratio of the composite material was assessed through energy dispersive X-ray analysis coupled with scanning electron microscopy in five different regions of the sample; the obtained value was 89.8/10.2, i.e., very close to the theoretical one (90/10), with cerium ions being shown to be evenly distributed in the material (see Supplementary Materials, Figure S2C).

### 2.6. Characterization of Extract-Loaded Materials

To preserve the radical scavenger capacity and antimicrobial potential of the common sage extracts, chosen extracts were embedded into mesoporous inorganic matrices, such as titania and titania-ceria composite, as well as commercial MCM-41 mesoporous silica. Two ethanolic extracts, So(US)-1 and So(Conv)-4, as well as the hydroalcoholic So(Conv)-3 extract, were selected (because of their high phytocompound content) for embedding into mesoporous matrices by incipient wetness impregnation technique followed by vacuum evaporation of the solvent. The materials containing extract, denoted *extract@support*, were characterized by thermal analysis (DTA-TG) to evaluate the content of polyphenolic substances and FTIR spectroscopy to demonstrate the presence of polyphenols in the mesoporous matrix. Also, the antioxidant capacity and antibacterial potential of the materials were assessed.

The content of phenolic compounds loaded into mesopores of titania or titania-ceria supports, as determined by DTA-TG analysis (Supplementary Materials, Figure S3), was in the range of 18.3–20% (wt), while in the case of embedding into MCM-41 mesoporous silica, it ranged between 32.4 and 38.4% (wt) due to a higher total pore volume of the silica matrix compared to the titania-based supports (0.88 cm^3^/g versus 0.26–0.54 cm^3^/g).

**Figure 4:**
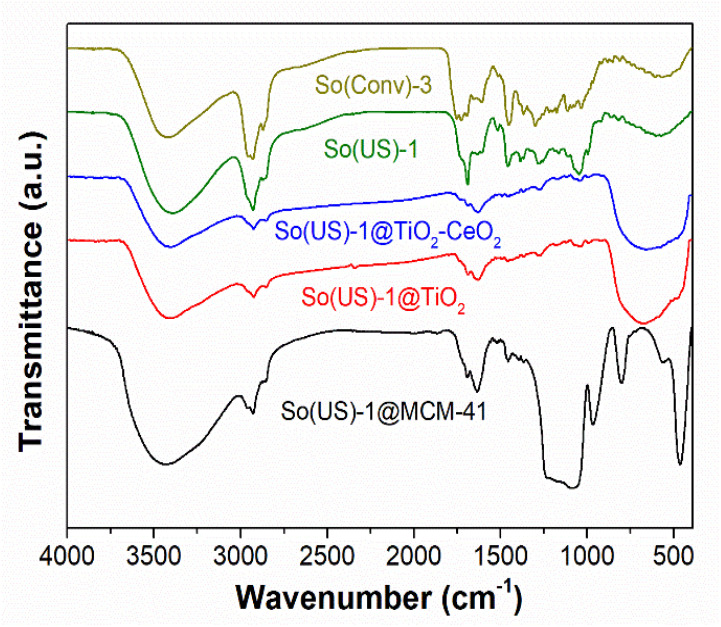
FTIR spectra of selected extracts and materials containing extract.

In the FTIR spectra of extract-loaded materials (Figure 4), one can observe the vibrations of both the mesoporous support and the polyphenolic compounds. For instance, in the case of So(US)-1@MCM-41, the bands at 1076 cm^−1^, 960 cm^−1^, 805 cm^−1^, and 459 cm^−1^ may be attributed to the silica support. The vibrations specific to the polyphenols are as follows: symmetric and asymmetric stretching vibrations of C-H bonds in the range of 2830–3000 cm^−1^; the stretching vibration of C=O bonds of carboxylic groups at 1753 cm^−1^ and 1728 cm^−1^ for hydroalcoholic So(Conv)-3 extract and ethanolic So(US)-1 sample, respectively; a band at 1694 cm^−1^, attributed to C=C bonds in aromatic rings; the intense vibrations at 1449 cm^−1^ and 1462 cm^−1^ for So(Conv)-3 and So(US)-1, respectively, attributed to deformation of the OH phenolic groups and C-O stretching vibrations; and the bands at 1107 cm^−1^ and 1047 cm^−1^ for So(Conv)-3 and So(US)-1, respectively, attributed to aromatic C-H deformation vibrations. In all extract-loaded samples, vibrations were observed in the range of 2830–3000 cm^−1^ and 1750–1270 cm^−1^, which evidenced the presence of polyphenolic compounds in the mesoporous inorganic matrices, and a large envelope in the range of 3200–3600 cm^−1^, attributed to the high content of OH groups in either phenolic compounds or mesoporous matrices. Also, in all embedded extracts, deformation vibrations of physically adsorbed water molecules were observed at around 1630 cm^−1^ [51].

### 2.7. Radical Scavenging Activity of Extract-Loaded Materials

The radical scavenger activity of the extract-loaded materials was evaluated through DPPH assay after 24 h of incubation in DPPH^•^ free radical solution in dark conditions, in duplicate, on solid samples. The results were compared with those of the free extract and support in the same quantity as that of the embedded extract using the degradation of DPPH^•^ solution as control. More details of this method are described elsewhere [30].

**Figure 5:**
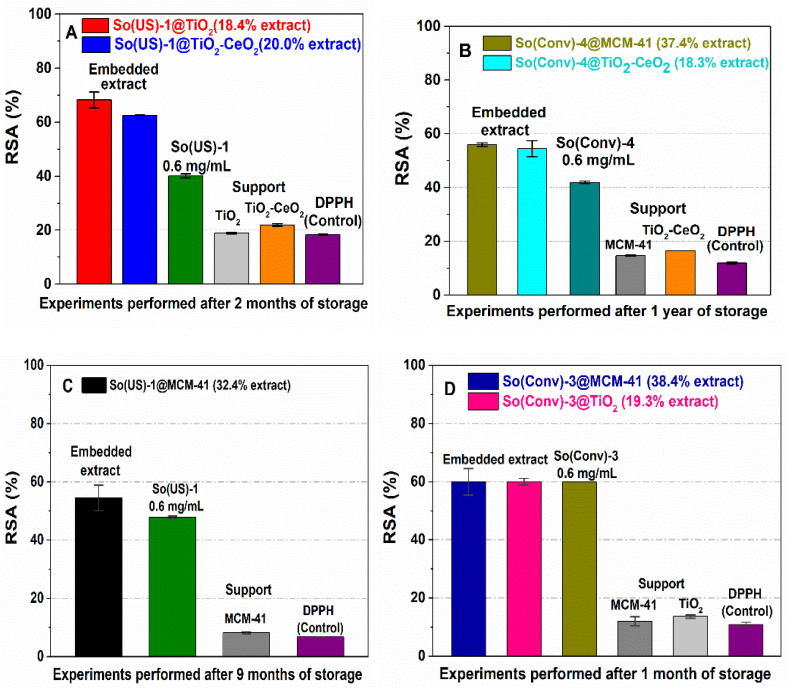
In vitro radical scavenger capacity of the ethanolic So(US)-1 extract embedded in TiO_2_ and TiO_2_-CeO_2_ mesoporous matrices (**A**), the ethanolic So(Conv)-4 extract embedded in MCM-41 silica and TiO_2_-CeO_2_ supports (**B**), So(US)-1@MCM-41 (**C**) and hydroalcoholic So(Conv)-3 embedded in TiO_2_ and MCM-41 matrices (**D**) in comparison with the corresponding contents of extract and support.

The extract-loaded materials exhibited a higher radical scavenger activity after 2–12 months of storage at 4 °C than the free extract (Figure 5), which means higher stability after embedding in a mesoporous support. The inorganic matrices used as supports did not make a noticeable contribution. However, TiO_2_-CeO_2_ material presented low radical scavenging activity due to the presence of a ceria phase, but a synergistic effect on the materials containing extract was not observed. For instance, an even lower RSA value was determined for So(US)-1@TiO_2_-CeO_2_ than for So(US)-1@TiO_2_ (Figure 5A), probably because a low amount of phenolic compounds was present on the surface of the support, and hence, the phytocompounds were not well protected. In the case of hydroalcoholic So(Conv)-3 extract loaded on either TiO_2_ support or MCM-41 silica matrix, after one month of storage, the radical scavenger capacity was the same as that of the free extract (Figure 5D). Our results are in agreement with literature data showing that the encapsulation of polyphenols in a matrix has the potential to preserve their antioxidant properties. For instance, Haładyn et al. showed that encapsulation in microspheres containing a combination of sodium alginate and guar gum significantly enhanced the stability of chokeberry polyphenolic extract, leading to the preservation of its antioxidant activity [52]. It has also been reported, based on in vivo tests, that *Salvia Officinalis* extract-loaded poly-lactic-co-glycolic acid nanoparticles are more effective than free extract against neurotoxic stress induced by methylmercury; this was attributed to the more efficient reduction of intracellular reactive oxygen species by the encapsulated extract compared to the free one [53].

### 2.8. Polyphenols Release Profiles from Mesoporous Titania and Silica Supports

**Figure 6:**
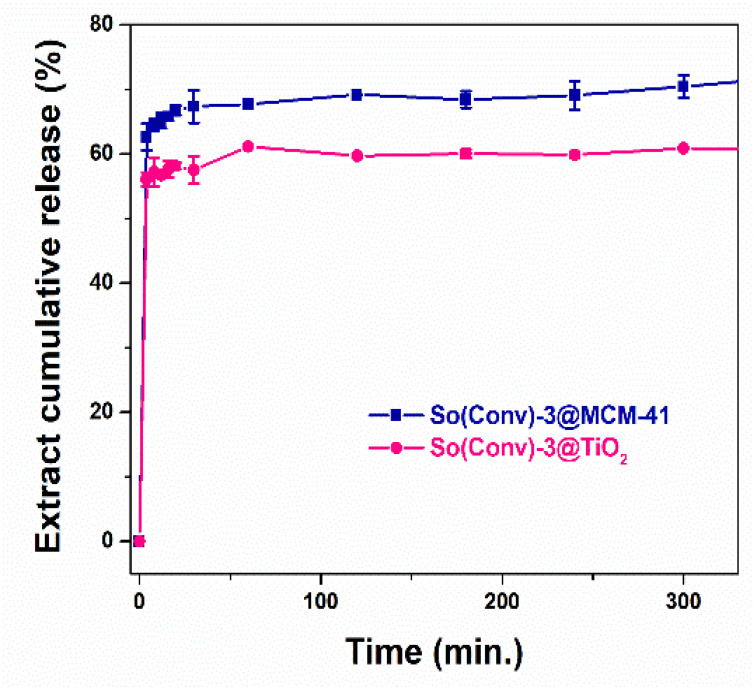
In vitro So(Conv)-3 extract release from titania and MCM-41 silica supports.

To study how the phenolic compounds from common sage hydroalcoholic extract are delivered from mesoporous titania and MCM-41 silica supports in simulated body fluid, extract release experiments were performed in PBS pH 5.7. A graph depicting the cumulative release of polyphenols over time is presented in Figure 6. In the case of both inorganic supports, a strong burst effect can be noticed in the first half hour, followed by a low quantity of polyphenol delivery over the next seven hours. A partial recovery of polyphenols from both supports was observed, with a slightly higher amount being released from MCM-41, 69.37 ± 1.19% than TiO_2_, 64.86 ± 2.58% (Figure 6), which showed slightly stronger interactions between phenolic compounds and the support. Recently, a combination of chemical and conductometric analysis was used to determine the release profile of the phytocompounds of five *Lamiaceae* plant extracts from alginate microbeads in distilled water at 23 °C. The highest values of conductivity were observed after 90 min for the sage microbeads, which means rapid phytocompounds release [54], in agreement with the observations reported here.

### 2.9. Bactericidal Activity of Extract-Loaded Materials

In order to assess the antibacterial activity of selected materials containing extract via the disk diffusion method, a gram-negative strain, i.e., *Pseudomonas aeruginosa* ATCC 27853, and a gram-positive strain, i.e., *Staphylococcus aureus* ATCC 25923, were chosen. The corresponding inhibition growth zone diameter, *Φ*, for each extract-loaded material in comparison with that of the free extract and the contribution of the corresponding support is presented in Table 5. The inhibition zone diameters of the materials containing embedded extract (*EM*) indicated the preservation of antibacterial activity after loading into the inorganic mesoporous matrix. Slightly larger inhibition zone diameters were obtained against *S. aureus*, probably due to differences between the bacterial cell wall structures. Also, a small improvement in bactericidal activity was observed for the So(US)-1 extract when loaded onto TiO_2_ material, in comparison with MCM-41 silica, because of the contribution of the support (Table 5). Typically, the encapsulation of plant extracts, including common sage, in polysaccharide (alginate, chitosan and starch) particles and liposomes, preserves the antimicrobial activity of bioactive compounds, e.g., against Gram-positive and Gram-negative bacteria; an improved bactericidal potential could be achieved only by co-encapsulation of the plant extract and lysozyme [55].

## 3. Materials and Methods

### 3.1. Materials

All substances for spectrometric determinations, i.e., Folin-Ciocalteu reagent (Sigma-Aldrich Co., Merck Group, Darmstadt, Germany), sodium carbonate (Sigma-Aldrich, ≥99.5%), aluminium chloride hexahydrate (Sigma-Aldrich, 99%), ethanol absolute (≥99.9% Supelco Inc., Belfonte, Kansas City, MO, USA), 6-hydroxy-2,5,7,8-tetramethylchroman-2-carboxylic acid (Trolox, Aldrich, 97%, Aldrich Chemical Co Inc., Milwaukee, WI, USA), 2,2-diphenyl-1-picrylhydrazyl (DPPH, Sigma-Aldrich), 2,2′-azino-bis(3-ethylbenzothiazoline-6-sulphonic acid) (ABTS, Sigma-Aldrich), and potassium persulphate (K_2_S_2_O_8_; Sigma-Aldrich, ≥99%), were used without additional purification.

For the high performance liquid chromatography (HPLC) analyses, the following standard compounds were used: protocatechuic acid (TCI, >98%, HPLC-grade), caftaric acid (Molekula GmbH, Munich, Germany), caffeic acid (Sigma, 98%, HPLC-grade), chlorogenic acid (HWI group, primary reference standard), rosmarinic acid (Sigma, >98%, HPLC-grade), gallic acid (Alfa Aesar, Ward Hill, MA, USA, 98%), catechin hydrate (Sigma, >98%, HPLC-grade), ellagic acid dihydrate (Tokyo Chemical Industry, Japan, TCI, >98%, HPLC-grade), chicoric acid (TCI, >98%), *trans*-ferulic acid (TCI, >98%, GC), vanillic acid (TCI, >98%, GC-grade), syringic acid (Molekula, >98.5%), (−) epicatechin (TCI, >98%, HPLC-grade), quercetin (Sigma, >95%, HPLC-grade), rutin hydrate (Sigma, 95%, HPLC-grade), *trans*-*p*-coumaric acid (Sigma Aldrich), myricetin (Sigma, >96%, HPLC-grade), *trans*-resveratrol (Sigma Aldrich, certified reference material), kaempferol (Sigma, >97%, HPLC-grade), cyanidin chloride (Sigma, >95%, HPLC-grade), malvidin chloride (Sigma Aldrich, >95%, HPLC), pelargonidin chloride (Aldrich) and delphinidin chloride (Sigma Aldrich, analytical standard), and for mobile phases acetonitrile (ACN, Riedel-de Haën, Honeywell Riedel-de Haën, Seelzer, Germany), and formic acid (Merck Group, Darmstadt, Germany). For all aqueous solutions preparation, extraction and analyses, ultrapure water (Millipore Direct- Q3 UV product no. C9185, Merck Group, Darmstadt, Germany), obtained via a water purification system with a Biopack UF cartridge, was used.

For the synthesis of mesoporous titania and titania-ceria composite materials, the following reagents were used as received: titanium(IV) isopropoxide (>97%, Aldrich), poly(ethylene glycol)-block-poly(propylene glycol)-block-poly(ethylene glycol), MW = 5800 (Pluronic, P123, Aldrich), acetic acid (>99.7%, Sigma-Aldrich Co. Merck Group, Darmstadt, Germany), cerium chloride heptahydrate (≥98%, Sigma-Aldrich), 2-propanol anhydrous (99.5%, Sigma-Aldrich), ethanol absolute (≥99.9% Supelco), and 25% (wt) ammonia aqueous solution (Scharlau). Also, commercial MCM-41 mesoporous silica (Sigma-Aldrich) was used.

### 3.2. Preparation and Characterisation of Common Sage Extracts

Dried leaves of *Salvia officinalis L.*, purchased from a local vendor, were chosen as the plant material. The ethanolic and hydroalcoholic (ethanol-water 1/1 *v*/*v*) polyphenolic extracts from *Salvia officinalis* L. were prepared at different plant/solvent weight ratios, at reflux or 50 °C, either by the conventional method or by microwave (MW power of 75 W; Milestone Technologies Neos Essential Oil Systems, CT, USA) or ultrasound (Bandelin Sonorex Digitec ultrasonic bath, Berlin, Germany)-assisted extraction in three extraction stages, with separation of the vegetal material after each stage and replacement of the solvent in the same volume, followed by pooling together of the three extract fractions. For the preparation of all extracts, a 20 h maceration step in the extraction solvent was applied. In the case of conventional extraction, each extraction stage lasted 1 h, while for MW or UAE, these were 15 min. The conditions applied for extract preparation and labeling are listed in Table 1.

The solvent was evaporated using a rotary vacuum evaporator DLAB RE100-Pro (DLAB Scientific, Beijing, China) until the extract reached a constant mass. It was redissolved to prepare extracts of known concentration.

The prepared polyphenolic extracts were characterized by spectrophotometric methods (Shimadzu UV-1800, Shimadzu Corporation, Kyoto, Japan) to assess the total polyphenol content using Folin-Ciocalteu reagent based on a calibration curve of gallic acid, total flavonoid content using aluminum chloride and expressed as quercetin equivalents, and total chlorophyll pigment content, calculated based on the Ritchie’s equations using solution absorbance values at 665 nm, 649 nm, and 750 nm. All methods applied for these spectrometric determinations, as well as the assessment of the radical scavenger activity of polyphenolic extracts by in vitro ABTS and DPPH assays, including the standard curves and the concentrations domain, have been described elsewhere [5]. The chemical profile of polyphenolic extracts was evaluated by reversed-phase high performance liquid chromatography with a photodiode array detector, i.e., HPLC-PDA (Shimadzu Nexera 2 with SPD-M30A detector, Shimadzu Corporation, Kyoto, Japan), operating in the 250–600 nm wavelength range. A Nucleoshell^®^ reversed-phase C18 column 4.6 × 100 mm (2.7 µm) (Macherey-Nagel GmbH & Co. KG, Düren, Germany), two mobile phases, i.e., 2.5% aqueous formic acid solution (mobile phase A) and 90% aqueous acetonitrile with 2.5% formic acid (mobile phase B), a gradient elution at constant flow of 0.4 mL/min at constant temperature of 20 °C, and 1 µL for the injection were used for the chromatographic analysis. The spectrophotometric methods and HPLC analysis have been described elsewhere [30]. For all experiments, the samples were weighed using a semimicro balance Precisa EP 225SM-DR (±0.01 mg) (Precisa Gravimetrics AG, Dietikon, Switzerland).

### 3.3. Obtaining Mesoporous Titania and Titania-Ceria Composite Supports

Mesoporous titania and titania-ceria composite materials were synthesized by the sol-gel method using titanium isopropoxide and cerium chloride heptahydrate as metallic sources in the presence of a structure directing agent, i.e., triblock copolymer Pluronic P123. Thus, for titania nanoparticles synthesis, 1.25 g of Pluronic P123 was dissolved in a mixture of 50 mL of anhydrous 2-propanol and 1.5 mL of glacial acetic acid, followed by the addition of 3.76 mL titanium isopropoxide under magnetic stirring. The reaction mixture was stirred at 40 °C for 1 h. Then, for hydrolysis and condensation reactions of titania species, 1 mL of water was added dropwise. The reaction mixture was first aged under magnetic stirring at 40 °C for 24 h and then solvothermal treated in a Teflon-lined autoclave under autogenous pressure at 100 °C for 24 h. After solvothermal treatment, the resulting white solid was separated by centrifugation, washed with ethanol and water, and dried at 100 °C. To remove the structure directing agent, Soxhlet extraction was performed in ethanol for 24 h, followed by a calcining step at 400 °C, 3 h with 0.5 °C/min heating rate.

The ceria-titania composite was obtained by dissolving Pluronic P123 (1.25 g) in anhydrous 2-propanol (50 mL) acidified with glacial acetic acid (1.5 mL), adding the appropriate volume of titanium isopropoxide (3.38 mL) and cerium chloride heptahydrate previously dissolved in 3 mL ethanol to the solution containing the template agent under magnetic stirring at room temperature. The reaction mixture was kept under reflux for 20 h, and then 2M aqueous NH_3_ solution was added dropwise to raise the pH to 9 in order to precipitate the cerium ions. Next, the reaction mixture was aged under magnetic stirring for another 24 h at 40 °C. The solid was filtered off and intensively washed with water and ethanol. Next, Soxhlet extraction was performed in ethanol for 24 h to remove most of the template agent. Finally, the composite material was calcined at 450 °C for 5 h with a heating rate of 0.5 °C/min.

### 3.4. Loading of Salvia Officinalis Extracts into Mesoporous Inorganic Matrices

The materials containing embedded extract were obtained by the incipient wetness impregnation method using So(US)-1 and So(Conv)-4 extracts and titania, titania-ceria composite and commercial MCM-41 as supports. Specifically, the mesoporous inorganic matrix was first dried in vacuum at 110 °C for 12 h, and then mixed with the selected common sage extract (20 mg/mL concentration). The resulting suspension was then dried in vacuum at room temperature for 12 h in dark conditions. The resulting materials containing extract were labelled *extract@support*.

### 3.5. Characterization of Inorganic Matrices and Materials Containing Extract

The mesoporous titania and titania-ceria composite supports were characterized by wide-angle X-ray diffraction (XRD), Fourier transform infrared spectroscopy (FTIR), thermal analysis (DTA-TG), and scanning and transmission electron microscopy. The FTIR spectra were recorded on a Bruker Tensor 27 spectrophotometer (Bruker Corporation Optik GmbH, Bremen, Germany) in the 4000–400 cm^−1^ domain. Wide-angle XRD analyses were recorded on a Rigaku MiniFlexII diffractometer (Rigaku Corporation, Tokyo, Japan) using Cu-Kα radiation in the 10°–70° 2*θ* range with a step of 0.01° and a scanning rate of 1°/min. SEM investigation of mesoporous inorganic materials was performed on a Tescan Vega 3 LM electron microscope (Tescan, Brno, Czech Republic) equipped with an energy dispersive X-ray (EDX) detector for chemical composition determination, while TEM investigation was carried out on a FEI (Hillsboro, Oregon, USA) TECNAI F30 G^2^ S-TWIN high resolution transmission electron microscope with a field emission electron gun and a maximum accelerating voltage of 300 kV. The nitrogen adsorption-desorption isotherms were recorded on a Quantachrome Autosorb iQ_2_ gas sorption analyzer (Quantachrome Instruments, Boynton Beach, FL, USA) at 77 K in order to determine the specific surface area by Brunauer-Emmett-Teller method in the relative pressure range of 0.05–0.25, the total pore volume measured at 0.99 relative pressure, and the average pore diameter by Barrett Joyner Halenda model. Before recording the isotherms, the inorganic supports were outgassed at 150 °C for 12 h. Thermogravimetric analyses were performed in synthetic air flow with a scan rate of 10 °C/min on a Mettler Toledo GA/SDTA851e (Mettler Toledo, Greifensee, Switzerland).

### 3.6. Antibacterial Activity Assessment of S. officinalis Extracts

The antibacterial activity of *Salvia officinalis* extracts was assessed against *Escherichia coli* ATCC *25922*, *Pseudomonas aeruginosa* ATCC 27853, and *Staphylococcus aureus* ATCC 25923 (Thermo Fisher Scientific-Waltham, MA, USA).

#### 3.6.1. Minimum Inhibitory Concentration (MIC)

The antibacterial properties of the tested polyphenolic extracts were determined by broth microdilution assay. The So(US)-1 and SO(Conv)-6 extracts were tested against three bacterial strains: *Escherichia coli* ATCC 25922, *Pseudomonas aeruginosa* ATCC 27853 and *Staphylococcus aureus* ATCC 25923. The common sage extracts were two-fold diluted in 96-well plate starting from 7 mg/mL extract concentration using TSB (Tryptic Soy Broth) medium. Each microtiter well was inoculated with microbial suspension with 1.5 × 10^8^ CFU/mL (0.5 McFarland) density. The minimum inhibitory concentration (MIC) values were determined after 24 h incubation at 37 °C by spectrophotometric evaluation of the optical density at 620 nm in each well. The solvent was used as control for the assessment of bacterial growth. Experiments for MIC determination were performed in duplicate. For each antimicrobial test, a sterility control of the TSB medium and a positive microbial growth control were used.

#### 3.6.2. Minimum Biofilm Eradication Concentration (MBEC)

The 96-well plates used for MIC assessment were drained and rinsed three times with sterile saline solution. The biofilm in each well was fixed with cold methanol for 20 min, followed by coloration with 1% violet solution for 15 min. After the removal of the violet crystal solution, the microplates were washed with water and the microbial biofilm was resuspended in 33% acetic acid solution. Microbial cell density was determined spectrophotometrically at 490 nm. The lowest extract concentration that inhibited the development of the microbial biofilm was considered the minimum biofilm eradication concentration (MBEC) value [56].

### 3.7. Antibacterial Activity of Materials Containing Extract through Disk Diffusion Method

Composite materials containing So(US)-1 extract embedded in inorganic carriers (mesoporous silica and titanium dioxide) were tested against *Pseudomonas aeruginosa* ATCC 27853 and *Staphylococcus aureus* ATCC 25923 via the disk diffusion method. Bacterial suspensions with 0.5 McFarland density were obtained from solid culture and used to inoculate Petri dishes containing agar medium. Subsequently, the composite materials were suspended in ethanol to achieve a final extract concentration of 14 mg/mL. A volume of 10 μL suspension was placed on the surface of the inoculated agar medium, which was then incubated at 37 °C for 18–24 h. The antibacterial activity was determined by measuring the bacterial growth inhibition zone diameter. The corresponding carrier was also tested against the chosen bacterial strains. Ethanol was used as control.

### 3.8. Determination of Polyphenols Release Profiles from Mesoporous Supports

In vitro release experiments of polyphenols from mesoporous supports were performed in 0.2 M phosphate buffer solution (PBS), pH 5.7, under constant magnetic stirring (200 rpm) at 37 °C in dark conditions, with a constant volume of release fluid. Each material containing polyphenolic extract (corresponding to 10 mg extract) was suspended in 20 mL PBS. At predetermined time intervals, aliquots of 100 µL were withdrawn from the release medium, diluted with PBS, and then analyzed by ultraviolet-visible spectrometry at 285 nm, corresponding to the determination of the total polyphenolic index according to a method described elsewhere [31]. The amount of recovered extract was determined based on a calibration curve for So (Conv)-3 extract in PBS pH 5.7 (*y* = 0.0096*x, R^2^ = 0.9998) in the concentration domain of 1–100 µg/mL.

## 4. Conclusions

To ensure the stability of common sage extracts over time, two inorganic mesoporous materials, i.e., titania and titania-ceria, were prepared by the sol-gel method via novel methods and then used for the loading of selected extracts. The efficiency of extract loading into these two materials was compared with that of commercial mesoporous MCM-41 silica. UAE showed a better efficiency of polyphenol recovery from *Salvia officinalis* L. dried leaves than conventional or MW-assisted extraction. The extract-loaded materials exhibited enhanced radical scavenger activity compared to the free extract after at least two months of storage at 4 °C, which means better stability over time of phenolic compounds following embedding into a mesoporous matrix. It was also shown that the encapsulation of the extract into titania nanoparticles slightly improved its antimicrobial activity due to the contributions of the support material. This approach could be further employed to develop new formulations for polyphenolic extracts with antibacterial properties.

## Figures and Tables

**Table 1:** Extraction parameters, yield of the extraction process, total polyphenol, flavonoid, and chlorophyll content, as well as radical scavenger activity for common sage polyphenolic extracts.

Extract	Solvent, *T* (°C), Plant/Solvent (*w*/*v*)	E (%wt)	TPC (mgGAE/ g_extract_)	TFC (mgQE/ g_extract_)	TChC (mgCh/ g_extract_)	RSA_ABTS_ (mg TE/ g_extract_)	RSA_DPPH_ (mg TE/ g_extract_)	IC50% (mg/mL)
So(US)-1	ethanol/50 °C; 1/30	13.0	192.81 ± 5.43	24.35 ± 0.20	12.86 ± 0.61	245.68 ± 6.28	201.29 ± 16.36	1.35
So(MW)-2	50% ethanol/80 °C; 1/50	19.9	168.97 ± 1.57	26.52 ± 0.20	0.53 ± 0.01	232.32 ± 0.73	211.86 ± 4.45	1.28
So(Conv)-3	50% ethanol/50 °C; 1/30	31.7	145.40 ± 2.31	25.11 ± 0.49	0.57 ± 0.08	215.20 ± 4.22	298.34 ± 10.42	0.91
So(Conv)-4	ethanol/ 80 °C; 1/18	14.0	129.20 ± 5.59	36.98 ± 1.22	4.40 ± 0.40	128.89 ± 4.80	249.44 ± 11.55	1.09
So(Conv)-5	50% ethanol/80 °C; 1/18	24.5	165.52 ± 2.99	23.62 ± 0.06	4.19 ± 0.15	249.07 ± 6.93	268.11 ± 11.22	1.01
So(Conv)-6	ethanol/50 °C; 1/30	8.2	138.11 ± 2.45	15.42 ± 0.11	3.56 ± 0.12	113.36 ± 2.40	98.22 ± 8.72	2.76

E-extract; TPC-total polyphenols content as gallic acid equivalents (GAE); TFC-total flavonoids as quercetin equivalents (QE); TChC–total chlorophyll pigments content, RSA-radical scavenger activity, TE-Trolox equivalents; IC50%–the extract concentration, which determines the inhibition of 50% of DPPH^•^ radicals from solution.

**Table 2:** Quantification of polyphenolic substances identified in the extracts by reverse phase HPLC-PDA.

Concentration in Extract (mg/g Extract)						
Compound	So(US)-1	So(MW)-2	So(Conv)-3	So(Conv)-4	So(Conv)-5	So(Conv)-6
protocatechuic acid	nd	0.571 ± 0.003	0.235 ± 0.007	nd	0.569 ± 0.014	nd
caftaric acid	0.760 ± 0.000	0.587 ± 0.001	nd	nd	0.746 ± 0.003	nd
chlorogenic acid	0.330 ± 0.000	0.675 ± 0.000	0.828 ± 0.004	1.028 ± 0001	0.753 ± 0.005	0.094 ± 0.000
caffeic acid	0.552 ± 0.000	2.494 ± 0.019	2.175 ± 0.000	0.874 ± 0.003	2.632 ± 0.000	0.174 ± 0.001
rosmarinic acid	35.335 ± 0.000	14.861 ± 0.008	22.877 ± 0.004	26.618 ± 0.063	20.542 ± 0.009	5.673 ± 0.025

nd–not detected.

**Table 3:** MIC and MBEC for common sage extracts.

Extract	MIC (µg/mL)			MBEC (µg/mL)	
	*P. aeruginosa*	*E. coli*	*S. aureus*	*P. aeruginosa*	*E. coli*
So(US)-1	875	1750	437.5	437.5	437.5
So(Conv)-6	875	1750	875	437.5	437.5

**Table 4:** Textural parameters of supports and the quantity of embedded extract in each support.

Support	d_BJH_ (nm)	S_BET_ (m^2^/g)	*V_p_* (cm^3^/g)	Embedded Extract	Extract (% wt)
TiO_2_	7.43	124	0.26	So(US)-1@TiO_2_	18.4
				So(Conv)-3@TiO_2_	19.3
TiO_2_-CeO_2_	13.18	150	0.54	So(US)-1@TiO_2_-CeO_2_	20.0
				So(Conv)-4@TiO_2_-CeO_2_	18.3
MCM-41	2.67	976	0.88	So(US)-1@MCM-41	32.4
				So(Conv)-4@MCM-41	37.4
				So(Conv)-3@MCM-41	38.4

**Table 5:** Inhibition growth zone diameter for materials containing extract.

	*Φ*, mm					
Sample	*P. aeruginosa* ATCC 27853			*S. aureus* ATCC 25923		
	*E*	*EM*	*S*	*E*	*EM*	*S*
So(US)-1@TiO_2_	14	15	14	16	17	11
So(US)-1@MCM-41	14	14	13	16	16	10

*E*-extract, *EM*-material containing embedded extract, *S*-support.

## Supplementary Materials

The following are available online at https://www.mdpi.com/article/10.3390/ma14216457/s1, Figure S1. Titania nanoparticles characterization: FTIR spectra of TiO_2_E and TiO_2_ (B); DTA curves of TiO_2_E and TiO_2_ (A); thermogravimetric curves of TiO_2_E and TiO_2_ (C); Figure S2. Titania-ceria composite characterization: FTIR spectra of TiO_2_-CeO_2_E and TiO_2_-CeO_2_ (B); DTA-TG curves of TiO_2_-CeO_2_E (A); DTA-TG analysis of TiO_2_-CeO_2_E; EDX elemental mapping image of TiO_2_-CeO_2_ composite material (C); Figure S3. DTA-TG analyses of the So(US)-1 extract embedded into MCM-41 and TiO2-CeO2 support.
