# Supplementary material for: Mesoporous Silica and Titania-Based Materials for Stability Enhancement of Polyphenols
Source: Materials (Basel). 2021 Oct 28;14(21):6457. doi: 10.3390/ma14216457 (PMC8585155; doi:10.3390/ma14216457)
Supplement: Supplementary file 1 [file materials-14-06457-s001.zip › materials-1421477-supplementary.pdf]

Mesoporous silica and titania-based materials for stability enhancement of polyphenols

Mioara Prundeanu[1], Ana-Maria Brezoiu[1], Mihaela Deaconu[1], Gratiela Gradisteanu Pircalabioru[2], Daniel Lincu[1,3], Cristian Matei[1] and Daniela Berger[1,*]

[1] Department of Inorganic Chemistry, Physical-Chemistry and Electrochemistry, University “Politehnica” of Bucharest, 1-7 Gheorghe Polizu Street, 011061, Bucharest, Romania; mioara_prundeanu@yahoo.com (M.P.), anamaria.brezoiu@gmail.com (A.-M.B.), mihaela_deaconu@yahoo.com (M.D.), cristi_matei@yahoo.com (C.M.)

[2] Research Institute of the University of Bucharest, Earth, Environmental and Life Sciences, Section-ICUB, 91–95 Splaiul Independenței, 050095, Bucharest, Romania; gratiela.gradisteanu@icub.unibuc.ro

[3] “Ilie Murgulescu” Institute of Physical Chemistry, Romanian Academy, 202 Splaiul Indepedentei, 060021, Bucharest, Romania; daniel.lincu1113a@gmail.com

* Correspondence: danaberger01@yahoo.com

1. *Characterization of Mesoporous Supports*

In the FTIR spectra of the titania sample obtained after the Soxhlet extraction process, TiO_2E, one can notice the characteristic bands of methyl and methylene groups in the rage of 2850-2930 cm^{-1} and 1410-1530 cm^{-1} proving the presence of the copolymer traces on the surface of TiO_2 NPs (Figure S1A-blue curve). In the FTIR spectrum of TiO_2 obtained at 400 °C, one can notice the following vibrations: 670 cm^{-1} (ν_{Ti-O}), 478 cm^{-1} ($\nu_{Ti-O-Ti}$), 3000-3600 cm^{-1} (ν_{Ti-OH}) assigned to TiO_2 NPs, and 1645 cm^{-1} (δ_{HOH}) attributed to physically adsorbed water molecules (Figure S1 A - black curve).

In agreement with FTIR spectroscopy, the DTA-TG analysis (Figure S1B and S1C) showed that the thermal treatment at 400 °C for 3h completely removed the copolymer as no effect was recorded on DTA curve of calcined titania sample. The copolymer decomposes in steps up to 350 °C. The extraction process was not very efficient, a content of 10% (wt.) copolymer remained in the TiO_2E material.

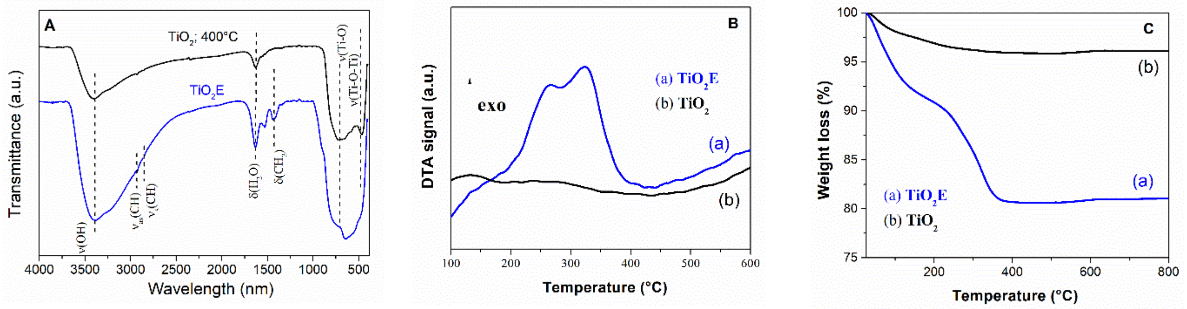

Figure S1. Titania nanoparticles characterization: FTIR spectra of TiO_2E and TiO_2 (B); DTA curves of TiO_2E and TiO_2 (A); thermogravimetric curves of TiO_2E and TiO_2 (C).

In the FTIR spectrum of TiO_2-CeO_2 composite recovered after the extraction process, one can observe the vibrations of the copolymer used as template agent in the range of 2859-2927 cm^{-1} ascribed to the stretching vibrations of methylene groups and 1430 cm^{-1} attributed to the deformation band of methylene groups (Figure S2-blue curve) that disappear in the FTIR spectrum of TiO_2-CeO_2 composite calcined at 450 °C, 5h, in which can be noticed the band from 3395 cm^{-1} assigned to the stretching vibrations of hydroxyl groups associated through hydrogen bonds present on particles surface, the shoulder at 3236 cm^{-1} ascribed to Ti-OH groups, besides the very intense bands centered at 667 cm^{-1} and 484 cm^{-1} attributed to the metal-oxide bonds (Figure S2A- black curve) that are shifted in comparison with Ti-O vibrations observed in the case of TiO_2 NPs (713 cm^{-1} and 460 cm^{-1}) (Figure S2B).

The calcination conditions for TiO_2-CeO_2 composite material were established based on DTA-TG analysis of material purified by an extraction step, TiO_2-CeO_2E, which showed the loss of adsorbed

water up to 120 °C corresponding to the first endothermic event on the DTA curve, followed by a weight loss of 4.9 % in the temperature range of 120 °- 450 °C assigned to the polymer burning (Figure S2B). The mapping analysis of titania-ceria composite showed a very uniform distribution of cerium ions in the material (Figure S2C).

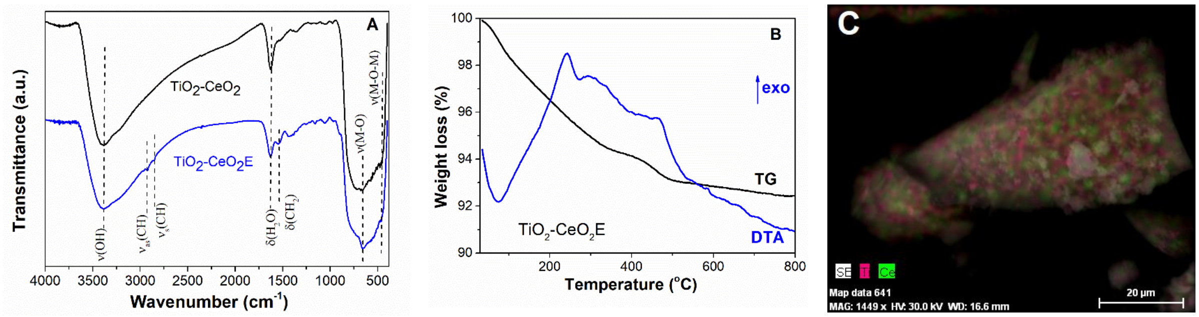

Figure S2. Titania-ceria composite characterization: FTIR spectra of TiO_2-CeO_2E and TiO_2-CeO_2 (B); DTA-TG curves of TiO_2-CeO_2E (A); DTA-TG analysis of TiO_2-CeO_2E; EDX elemental mapping image of TiO_2-CeO_2 composite material (C).

2 Characterization of Extract-Loaded Materials

The content of polyphenols in materials containing extract was determined by DTA-TG analysis based on the total weight loss up to 500 °C (in the case of the samples containing TiO_2 or TiO_2-CeO_2) or 600 °C (when MCM-41 was used as support), after subtraction of the weight loss attributed to the physically adsorbed water molecules that corresponds to the first endothermic event on DTA curve (Figure S3). One can notice that the thermal decomposition of phytocompounds took place at lower temperature when the polyphenolic extract was entrapped into TiO_2-CeO_2 support than in the case of MCM-41 mesoporous silica, probably because of the catalytic effect of titania-ceria matrix or because the interparticle extract is more exposed to oxidation than inside the pores.

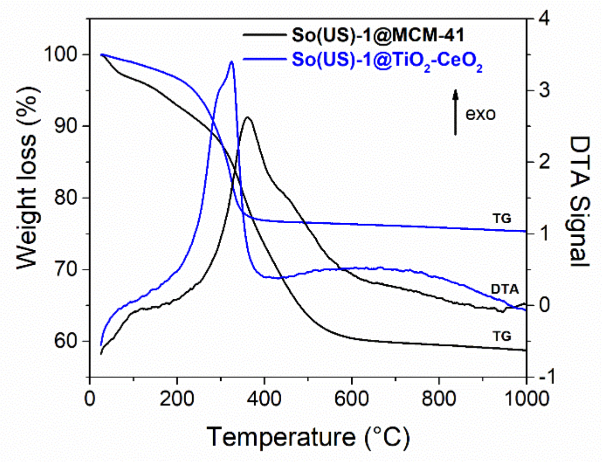

Figure S3. DTA-TG analyses of the So(US)-1 extract embedded into MCM-41 and TiO_2-CeO_2 support.
